# Chemotherapy-related hand–foot syndrome and hand–foot skin reaction: a review of management and possible approaches for Asian patients by the Japanese pharmacist-led oncodermatology study team

**DOI:** 10.1007/s10147-025-02895-y

**Published:** 2025-10-06

**Authors:** Yohei Iimura, Hirotoshi Iihara, Yoshitaka Saito, Hisanaga Nomura, Takuya Iwamoto, Mayumi Kotera, Yusuke Tsuchiya, Tatsuya Sumiya, Mariko Kono, Daisuke Hirate, Tomohiro Kurokawa, Toshinobu Hayashi, Hironobu Hashimoto, Junichi Higuchi, Ryuta Urakawa, Hiroyuki Saotome, Seiichiro Kuroda

**Affiliations:** 1 Department of Pharmacy, The IMSUT Hospital, The Institute of Medical Science, The University of Tokyo, 4-6-1, Shirokanedai, Minato-Ku, Tokyo, 108-8639 Japan; 2 Department of Pharmacy, Gifu University Hospital, Gifu, Japan; 3 Department of Clinical Pharmaceutics & Therapeutics, Faculty of Pharmaceutical Sciences, Hokkaido University of Science, Sapporo, Japan; 4 Department of Clinical Pharmacology and Therapeutics, Kyoto University Hospital, Kyoto, Japan; 5 Department of Pharmacy, Mie University Hospital, Tsu, Japan; 6 Department of Pharmacy, Ageo Central General Hospital, Ageo, Japan; 7 Department of Pharmacy, Yokohama City Minato Red Cross Hospital, Yokohama, Japan; 8 Department of Pharmacy, Tokyo Metropolitan Police Hospital, Tokyo, Japan; 9 Department of Pharmacy, Teine Keijinkai Hospital, Sapporo, Japan; 10 Department of Surgery, Jyoban Hospital of Tokiwa Foundation, Iwaki, Japan; 11 Department of Medical Epigenomics Research, Fukushima Medical University, Fukushima, Japan; 12 Department of Emergency and Disaster Medical Pharmacy, Faculty of Pharmaceutical Sciences, Fukuoka University, Fukuoka, Japan; 13 Department of Pharmacy, National Cancer Center Hospital, Tokyo, Japan; 14 Department of Pharmacy, Ohara Healthcare Foundation Kurashiki Central Hospital, Kurashiki, Japan; 15 Department of Pharmacy, The University of Osaka Dental Hospital, Osaka, Japan; 16 Department of Clinical Pharmacy Research and Education, Graduate School of Pharmaceutical Sciences, The University of Osaka, Osaka, Japan; 17 Department of Pharmacy Jyoban Hospital, Public Interest Foundation Tokiwakai, Iwaki, Japan

**Keywords:** Hand–foot syndrome, Hand–foot skin reaction, Chemotherapy, Molecular targeted therapy, Supportive care

## Abstract

**Background:**

Hand–foot syndrome (HFS) and hand–foot skin reaction (HFSR) are adverse effects induced by cytotoxic chemotherapeutic agents, such as capecitabine, pegylated liposomal doxorubicin, and multi-kinase inhibitors. HFS/HFSR can significantly reduce patients’ quality of life and impact cancer treatment intensity due to severe pain in the hands and feet. Although several recommendations and guidelines have been published, most focus on European and American populations, with no management guidelines specifically addressing Asian patients. Given that Asian skin types differ from those of Europeans and Americans, treatment recommendations tailored to Asian populations are needed.

**Methods:**

A narrative review of published articles retrieved following a systematic search of PubMed, the Cochrane Library, Medical Online, and Ichushi-Web between January 2000 and March 2025 was conducted. The search strategy targeted clinical trials using keywords related to HFS, palmar–plantar erythrodysesthesia, HFSR, prevention, therapy, and relevant anticancer agents. The review adhered to the PRISMA 2020 guidelines; However, formal quality assessment tools such as GRADE or the Cochrane risk-of-bias tool were not applied.

**Results:**

In total, 53 articles were included in this review, which found different recommendations from European countries due to the differences in skin type. Among the recommended treatments was topical diclofenac, suggested as a potential and novel prevention strategy for capecitabine-induced HFS. However, high potent topical steroids, such as lidocaine patches, or antiseptic solutions, were not recommended.

**Conclusions:**

This review provides evidence-based recommendations for the prevention and treatment of HFS/HFSR in Asian patients, taking into account their unique skin characteristics.

## Introduction

Hand–foot syndrome (HFS), also known as palmar–plantar erythrodysesthesia syndrome, is a cutaneous adverse event associated with cytotoxic agents (CTx) and BRAF inhibitors. HFS is characterized by pain, swelling, numbness, tingling, or hands and feet erythema, and was first described in association with mitotane administration [1]. Among chemotherapeutic agents, capecitabine and pegylated liposomal doxorubicin showed associations with a high severe skin toxicity incidence. Clinically significant HFS (grade ≥ 2), presenting with swelling, blistering, desquamation, or ulceration, can markedly impair patients’ quality of life (QOL) [2]. However, multi-kinase inhibitors (MKIs) are associated with hand–foot skin reaction (HFSR), a distinct but related dermatologic toxicity affecting the palms and/or soles [3]. Compared with HFS, HFSR typically presents earlier during treatment and is often of greater severity [4, 5].

The pathogenesis of HFS/HFSR differs. HFS is diffusely expressed in the palms and the soles, whereas HFSR is often localized in the areas of stress [6]. Additionally, by onset, HFSR may develop earlier than HFS. The mechanism involved HFS development differs from that of HFSR. Regarding HFS [6], (1) inhibition of proliferation of skin basal cells, (2) drug secretion from eccrine sweat glands, (3) involvement of drug degradation products, and (4) inflammatory response caused by interleukin (IL)−1α, IL-1β, IL-6, and reactive oxygen species have been suspected [7–10]. By contrast, HFSR is induced by the simultaneous inhibition of different receptors, which alters the structure of the microvasculature and causes abnormal endothelial and vascular repair mechanisms [11, 12]. Differential preventive and treatment strategies for HFS/HFSR are required because they are mutually exclusive. Because HFS has a disseminated onset, hands and feet should be cared for. In contrast, HFSR occurs in specific stressed or damaged areas, by any factor (traumatic or chemical stress). Therefore, intensive care is required in stress-prone areas.

Several evidenced-based clinical guidelines exist on patients in the United States and Europe [13, 14]. However, none are available for patients undergoing HFS or HFSR in Asia. Particularly, Asians are prone to developing HFS/HFSR caused by chemotherapeutic agents [15–17]. Therefore, Asian patients require suitable preventive and treatment strategies for HFS/HFSR. This study conducted an updated literature review and discussed possible management of HFS/HFSR.

## Methods

**Fig. 1:**
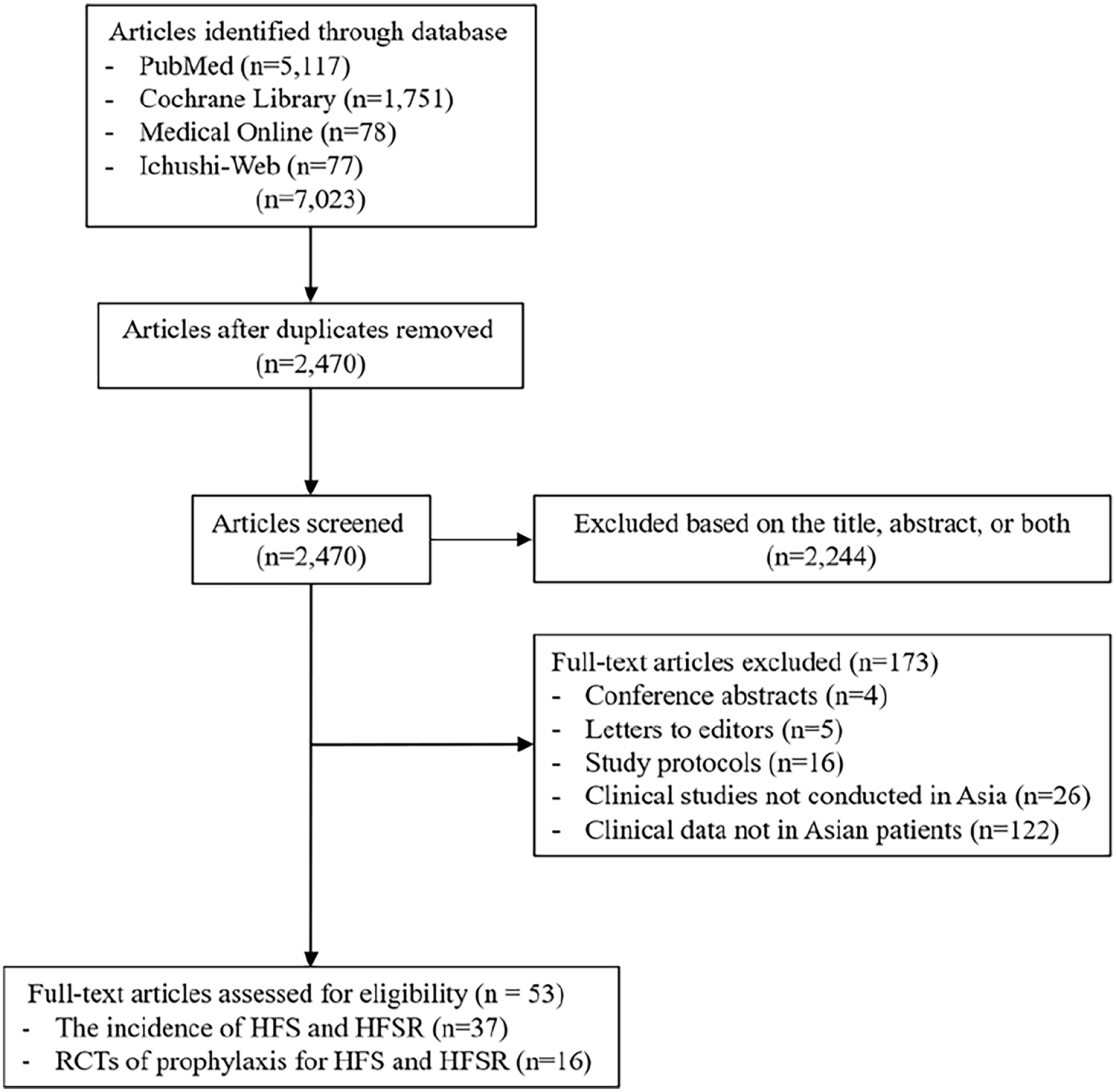
Diagram of study selection/screening process

In total, 7,023 articles published between January 2000 and March 2025 were identified through PubMed, the Cochrane Library, Medical Online, and Ichushi-Web searches. Only English language articles were included, and the selection was conducted by a single reviewer. The search strategy focused on clinical trials, using the following keywords: HFS, palmar-plantar erythrodysesthesia syndrome, HFSR, prevention, therapy, capecitabine, pegylated liposomal doxorubicin, doxorubicin, 5-fluorouracil, docetaxel, cytarabine, vemurafenib, dabrafenib, encorafenib, regorafenib, sorafenib, sunitinib, lenvatinib, cabozantinib, pazopanib, axitinib, and skin. After removing 2,470 duplicate records, 2,244 publications were excluded following the title and abstract screening, due to research topic irrelevance. A further 173 were excluded at full-text review, because they had no usable data. The literature screening process is summarized in Fig. 1. This review was narrative in nature, and study inclusion was determined primarily by relevance rather than strict methodological quality. The methodology and reporting of this review followed the Preferred Reporting Items for Systematic Reviews and Meta-Analyses (PRISMA) 2020 guidelines. Quality assessment tools such as GRADE or the Cochrane risk-of-bias tool were not applied.

## Results

Of the 53 included studies, data from 37 articles on the incidence of HFS and HFSR were extracted, and are summarized in Table 1.

**Table 1:** Incidence of HFS/HFSR according to cytotoxic agents in Asian patients

Cytotoxic agents	Incidence (%)
CTx inducing HFS	
Capecitabine	73.4– ≥ 90% [18–20]
Pegylated liposomal doxorubicin	15–78.4% [21–23]
Doxorubicin	22–26%, data are unavailable specifically for Asia [24]
5-fluorouracil	14–31% [25–27]
Docetaxel	6–41.2% [28, 29]
Cytarabine	14–33%, data are unavailable specifically for Asia [24]
BRAF inhibitors inducing HFS	
Vemurafenib	10–47.8% [33, 34]
Dabrafenib	42% [35], data are unavailable specifically for Asia
Encorafenib	5.1–27.3% [36, 37]
MKIs inducing HFSR	
Regorafenib	47–73% [38–40]
Sorafenib	4.5%–45% [41, 42]
Sunitinib	43–57.8% [43, 44]
Lenvatinib	27–40.5% [45, 46]
Cabozantinib	35.3–71.4% [47–50]
Pazopanib	3–14.8% [51, 52]
Axitinib	14.4–75% [53–57]

HFS, hand–foot syndrome; CTx, cytotoxic agent; HFSR, hand–foot skin reaction; MKIs, multi-kinase inhibitors

### Incidence of HFS according to cytotoxic agents

As Table 1 shows, CTx, that are capable of inducing HFS include capecitabine (73.4– ≥ 90% [18–20]), pegylated liposomal doxorubicin (15–78.4% [21–23]), doxorubicin (22–26%, data are unavailable specifically for Asia [24]), 5-fluorouracil (5-FU) (14–31% [25–27]), docetaxel (6–41.2% [28, 29]), and cytarabine (14–33%, data are unavailable specifically for Asia [24]). The incidence of grade ≥ 2 HFS (drug withdrawal should be considered) was reported to be up to 40% [18, 23, 30–32]. With regard to BRAF inhibitors, vemurafenib (10–47.8% [33, 34]), dabrafenib (42% [35], data are unavailable specifically for Asia), and encorafenib (5.1–27.3% [36, 37]) induce HFS.

### Incidence of HFSR according to cytotoxic agents

Also in Table 1, MKIs, including regorafenib (47–73%[38–40]), sorafenib (4.5%–45% [41, 42]), sunitinib (43–57.8% [43, 44]), lenvatinib (27–40.5% [45, 46]), cabozantinib (35.3–71.4% [47–50]), pazopanib (3–14.8% [51, 52]), and axitinib (14.4–75% [53–57]) induced HFSR.

### Randomized controlled trials (RCTs) of prophylaxis for HFS

Of the 53 included studies, data from 11 articles on RCTs of prophylaxis for preventing HFS/HFSR were extracted and are summarized in Table 2.

**Table 2:** Randomized controlled trials of prophylaxis for HFS

Country, year	Prophylaxis	Blinding	Population (N)	Chemotherapy	Intervention	Comparator	Outcomes
China 2010 [30]	Celecoxib	No	*N* = 101 Stage II–III colorectal cancer	Capecitabine-based	*N* = 51 Celecoxib 200 mg BID daily with capecitabine-based chemotherapy (monotherapy or CAPOX)	*N* = 50 Without celecoxib	Celecoxib reduced the incidence of grade > 2 HFS (11.76% vs. 30% *P* = 0.024)
China 2012 [18]	Celecoxib	No	*N* = 150 Stages II–III colorectal cancer	Capecitabine-based	*N* = 68 Celecoxib 200 mg BID daily with capecitabine-based chemotherapy (monotherapy or CAPOX)	*N* = 71 Without celecoxib	Celecoxib reduced the incidence of grade 2 HFS (14.7% vs. 29.6% *P* = 0.035)
Iran 2023 [64]	Celecoxib topical hydrogel	No Self-controlled	*N* = 28	5-fluorouracil, capecitabine, docetaxel, and paclitaxel	*N* = 28 one-half to 1 teaspoon of the celecoxib hydrogel BID	–	No significant difference in all grade (*P* = 0.38)
South Korea 2010 [88]	Pyridoxine	Yes	*N* = 389 Gastrointestinal tract cancers	Capecitabine	*N* = 180 Pyridoxine 100 mg BID	*N* = 180 Placebo	No significant difference in all grade HFS (64% vs. 76.1% *P* = 0.13) in first three chemotherapy
Japan 2014 [89]	Pyridoxine	No	*N* = 60 Colorectal cancer	Capecitabine	*N* = 30 Pyridoxine 60 mg/day	Without pyridoxine	No significant difference in grade ≥ 2 HFS (60.0% vs. 60.0% *P* = 1.00)
Singapore 2017 [90]	Pyridoxine	Yes	*N* = 210 Breast and colorectal cancers	Capecitabine	*N* = 105 Pyridoxine 200 mg/day	*N* = 105 Placebo	No significant difference in grade ≥ 2 HFS (31.4% vs. 37.1% *P* = 0.38)
Japan 2018 [91]	Pyridoxine	No	*N* = 133 Advanced or metastatic breast cancer	Capecitabine	*N* = 66 Pyridoxine 60 mg/day	*N* = 67 Without pyridoxine	No significant difference in grade ≥ 2 HFS (28.8% vs. 31.3% *P* = 0.75)
Japan 2020 [31]	Pyridoxine Eppikajutsuto (Kampo medicine)	No	*N* = 22 Colorectal cancer	Capecitabine-based	*N* = 10 Pyridoxine 20 mg TID	*N* = 12 Eppikajutsuto (Kampo medicine) 2500 mg	No significant difference in grade ≥ 2 HFS (40.0% in pyridoxine group, 50.0% in Eppikajutsuto group *P* = 0.22)
China 2021 [58]	Lithium contained topical and moisturizing	Yes	*N* = 122 Stages II-III colorectal cancer	Capecitabine	*N* = 51 EVOSKIN® Palm and Sole moisturizing cream BID	*N* = 54 Physiological saline BID	EVOSKIN® Palm and Sole moisturizing cream reduced the incidence of grade ≥ 1 and grade ≥ 3 HFS (56.8% vs. 75.9% P = 0.006, 6.0% vs. 18.5% *P* = 0.001, respectively)
India 2024 [65]	Diclofenac gel	Yes	*N* = 311 Breast and gastrointestinal cancer	Capecitabine	*N* = 130 Diclofenac 1% gel up to four times per day	*N* = 133 Placebo gel	Diclofenac gel reduced the incidence of grade ≥ 2 HFS (3.8% vs 15.0% *P* = 0.003)
India 2020 [32]	Structured Teaching Module	Yes	*N* = 280 Colorectal cancer	Capecitabine -based	*N* = 135 Continuous structured teaching module by oncology nurse	*N* = 134 Routine clinical education	No significant difference was found in the incidence of grade ≥ 2 HFS (33.3% vs. 32.8% *P* = 0.93)

HFS, hand–foot syndrome; CAPOX, capecitabine combined with oxaliplatin; N.D., not detected

### Clinical studies on the prevention of HFS

#### Moisturization

Only one RCT was found on HFS prevention by moisturization [58]. No other RCT examined the effects of moisturization alone.

#### Pyridoxine

Most RCTs on pyridoxine showed negative results, and multiple systematic reviews and meta-analyses have shown insufficient efficacy [59, 60].

#### Celecoxib

Two RCTs in China demonstrated preventive efficacy against HFS [18, 30]. However, an RCT in UK showed negative result [61]. Celecoxib is associated with a high risk of cardiovascular side effects [62, 63], gastrointestinal ulceration, and/or perforation, which may worsen with long-term prophylaxis. Efficacy of topical celecoxib hydrogels has not been demonstrated the efficacy in a self-controlled randomized study [64].

#### Topical diclofenac

The Diclofenac-topical for Reduction of capecitabine-related HFS (D-TORCH) study [65] suggested a preventive effect of diclofenac gel against capecitabine-induced HFS. However, in the D-TORCH study, the diclofenac gel was administered exclusively on the palms. Additionally, gels can induce skin dryness, leading to HFS and itchiness. Phase 3 RCTs evaluating the preventive effects of diclofenac cream on the palms and soles are ongoing in Japan [66].

#### Topical steroid

A phase 2 study evaluated hydrocortisone and reported its efficacy in capecitabine-induced HFS [67, 68]. However, no clinical recommendations exist regarding its prophylactic use as current evidence is insufficient to support the preventive effect of topical steroids on HFS. Regarding the recommended therapy, expert opinion suggests the use of high-potency topical steroids for patients with grade ≥2 HFS (Common Terminology Criteria for Adverse Events [CTCAE] v5.0) [69].

#### Topical antibiotics

Prophylactic evidence supporting the use of topical antibiotics, which are otherwise effective in treating symptoms associated with infections, is lacking. They may be considered for treating infections associated with HFS.

#### Cooling

Efficacy of cooling against HFS induced by pegylated liposomal doxorubicin has been demonstrated [70–72], but no designed trials exist. Oxaliplatin-induced peripheral neuropathy is exacerbated when cooling is performed in patients indicated for CAPOX therapy. Besides patients receiving oxaliplatin, cooling may be considered for preventing HFS.

#### Teaching program

Case–control and retrospective studies have revealed efficacy of teaching programs [73, 74]; however, RCT finding was negative [32].

#### Hydrocolloid dressing

Only one trial has been designed [75] and a phase 3 randomized self-controlled study is ongoing [76].

### RCTs of prophylaxis for HFSR

Of the 53 included studies, data from five articles reporting RCT findings on prophylaxis for preventing HFSR were extracted and are summarized in Table 3.

**Table 3:** Randomized controlled trials of prophylaxis for HFSR

Country, year	Prophylaxis	Blinding	Population (N)	Chemotherapy	Intervention	Comparator	Outcomes
China 2020 [81]	Celecoxib	No	*N* = 116 Advanced HCC	Sorafenib	*N* = 58 Celecoxib 200 mg BID	*N* = 58 Without celecoxib	Celecoxib reduced the incidence of grade ≥ 2 and grade 3 HFSR (29.3% vs. 63.8% *P* < 0.001, 3.4% vs. 19.0% *P* = 0.008, respectively)
China 2015 [78]	Urea cream	No	*N* = 871 Advanced HCC	Sorafenib	*N* = 439 10% urea cream TID + BSC	*N* = 432 BSC	10% urea cream T + BSC reduced the incidence of grade ≥ 2 HFSR (20.7% vs. 29.2% *P* = 0.004)
South Korea 2020 [79]	Urea cream	Yes	*N* = 288 Advanced HCC	Sorafenib	*N* = 130 20% urea cream TID	*N* = 117 Placebo	No significant difference in grade ≥ 2 HFSR (50.8% vs. 59.8% *P* = 0.153)
Taiwan 2022 [80]	Urea cream	Yes	*N* = 129 Advanced HCC	Sorafenib	*N* = 42 10% urea cream + BSC	*N* = 42 BSC *N* = 41 Moisturizing cream + BSC	No significant difference was found in the incidence density of HFSR in each groups (*P* > 0.05)
Japan 2013 [75]	Hydrocolloid dressing	No	*N* = 33 Advanced renal cell carcinoma	Sorafenib	*N* = 14 Hydrocolloid dressing containing ceramide with a low-friction external surface every 2–3 days	*N* = 16 10% urea cream	Hydrocolloid dressing reduced the incidence of grade ≥ 2 HFSR (29% vs. 69% *P* = 0.03) on the soles of the feet

HFSR, hand–foot skin reaction; HCC, hepatocellular carcinoma; BSC, best supportive care; N.D., not detected

### Clinical studies for the prevention of HFSR

#### Moisturization

No study that has evaluated the preventive effect of moisturization for HFSR has been reported. However, poor adherence to moisturizer use affects the therapeutic efficacy of regorafenib [77].

#### Urea cream

Urea cream use has been evaluated for its prophylactic efficacy in sorafenib-related HFSR [78–80]. Topical urea is effective against keratinized skin because it improves skin keratinization. However, its effectiveness shows mixed results. Urea cream can also induce skin irritation. While exercising caution regarding skin irritation, urea cream should be considered for the prevention of HFSR.

#### Celecoxib

One RCT in China demonstrated preventive efficacy against HFSR [81]. However, an RCT in UK showed negative results [61]. Celecoxib is associated with a high risk of cardiovascular side effects [62, 63], gastrointestinal ulceration, and/or perforation, which may worsen with long-term prophylaxis.

#### Topical steroid

Prophylactic effect of clobetasol on regorafenib-induced HFSR was previously reported [82]. However, no clinical recommendations exist regarding its prophylactic use as current evidence is insufficient to support its preventive effect on HFSR. Regarding the recommended therapy, expert opinion suggests the use of high-potency topical steroids for patients with grade ≥1 HFSR (CTCAE v5.0) [69].

#### Topical antibiotics

Prophylactic evidence supporting the use of topical antibiotics, which are otherwise effective in treating symptoms associated with infections, is lacking. They may be considered for treating infections associated with HFSR.

#### Hydrocolloid dressing

Only one trial has been designed [75] and a phase 3 randomized self-controlled study is ongoing [76].

### Therapeutic medication

To date, no clinical trial has evaluated the therapeutic effects of medications for managing HFS/HFSR. However, various guidelines recommend topical steroids for inflammation and pain, 10% urea cream for exfoliation, and topical antibiotics [13, 14]. These medications are essential for controlling symptoms in clinical practice. Therapeutic strategies should be considered following previous guidelines and recommendations.

## Discussion

### Possible management of HFS and/or HFSR.

#### HFS induced by CTx and BRAF inhibitors

High-level evidence exists for preventing capecitabine-related HFS using oral celecoxib and diclofenac. Oral celecoxib is associated with a high risk of cardiovascular side effects [62, 63], gastrointestinal ulceration, and/or perforation, which may worsen with long-term prophylactic use. Considering its safety, oral celecoxib has limitations as a preventive strategy. Limited prophylactic administration duration should be considered, and caution for adverse events. In contrast, topical diclofenac is relatively safe, and high-level evidence from the only large blinded RCT showed positive result [65]. However, in an RCT evaluating the preventive effect of diclofenac on capecitabine-related HFS (D-TORCH study) [65], HFS incidence in the placebo group was notably lower than for other Asian countries (Table 4). Differences in the frequency of all grades of HFS (> 30%) should be noted. Furthermore, diclofenac gel was administered only to the palms in the D-TORCH study, and its preventive effect on the soles remains unclear. To allay concerns about data reproducibility and effectiveness on soles, a phase 3 RCT evaluating the preventive effects of diclofenac cream on palms and soles is ongoing in Japan [83]. Regarding the prophylactic use of topical steroids, a subset analysis [84] showed no prophylactic efficacy in sorafenib-induced HFSR. However, results of a phase 2 trial currently investigating the prophylactic efficacy of topical hydrocortisone in capecitabine-induced HFS in Japan [67] are awaited. Stress avoidance and moisturization may be recommended because of their safety; however, no RCTs have evaluated their preventive effects. Instead, lithium-containing topical and moisturizing agents showed preventive effects against HFS in a well-designed study [58] although this study showed no preventive effect of moisturizers only; however, the study agent reduced incidence of grade ≥ 1 HFS. Regarding urea cream, preventive effect against grade ≥ 2 HFSR was shown [78]. Although urea cream irritates the skin in unfit patients, it can be therapeutically effective against HFSR, which is prone to keratinization. For grade ≥ 2 HFS/HFSR, topical steroid is essential to control skin discomfort and inflammation [13]. Since evidence is lacking, the therapeutic use of topical steroids should be limited at this stage. Various guidelines recommend high potent topical steroid therapy for grade ≥ 2 HFS/HFSR [13, 14], and such is used in clinical practice, although no RCTs have been conducted. If local infections are suspected, topical antibiotics are needed although their efficacy has not been evaluated in RCTs. Regarding teaching programs and hydrocolloid dressings, no recommendations exist introducing them clinically at this stage, and future trials are awaited.

**Table 4:** Randomized controlled trials showing differences in the incidence of HFS induced by capecitabine in India (D-TORCH study) and other Asian countries

			Incidence rate of HFS (any grade)		Incidence rate of HFS (grade 2 or 3)		Incidence rate of HFS (≥ grade 3)	
	Number of patients		%		%		%	
Country	Intervention	Control	Intervention	Control	Intervention	Control	Intervention	Control
China 2010 [30]	51	50	29	52	11.76	30	1.96	10
China 2012 [18]	68	71	57.4	74.6	14.7	29.6	2.9	8.5
South Korea 2010 [88]	180	180	64	76.1	12	15.4	3.3	5
Singapore 2017 [90]	105	105	61	66	32	37	4	2
Japan 2018 [91]	66	67	77	69	34	34	4	4
Japan 2020 [31]	10	12	70	83.3	40	50	20	8.3
China 2021 [58]	51	54	56.8	75.9	4.8	16.7	6.0	16.7
India 2020 [32]	135	134	64.4	62.5	33.3	32.8	5.2	6.8
India 2024 [65]	130	133	6.1	18.1	3.8	15	2.3	5.3

HFS, hand–foot syndrome

Table 5 summarizes possible management approaches. Managing HFS induced by CTx and BRAF inhibitors should be symptom grading-based according to CTCAE. Preventive measures (grade 0) include avoiding mechanical or chemical stress, limiting water exposure, wearing appropriately fitted gloves, regular use of emollients, and patient education. Topical diclofenac or short-term oral celecoxib may also be considered. For grade 1 (mild) HFS, observation, continuing same dose chemotherapy, moisturizing, and using keratolytic agents, such as 10% urea cream, are recommended. Grade 2 (moderate) HFS, which limits instrumental activities of daily living (IADL), requires very strong topical corticosteroids (to step-down following improvement), possible chemotherapy dose modification or postponement, and dermatology referral. For grade 3 (severe) HFS, which interferes with self-care, strongest corticosteroids are indicated, with chemotherapy interruption or dose reduction, topical antibiotics if infection is suspected, and mandatory dermatology consultation. Continuous patient education is emphasized across all grades.

**Table 5:** Management of HFS induced by CTx and BRAF inhibitors

Grading of CTCAE	Management	Follow up
Grade 0 prevention	Avoid stress mechanical and chemical stress (excessive exercise and external damage) should be avoided Excessive exposure to water should be avoided and gloves are recommended Gloves that are tight in size are not recommended Moisturizing Apply evenly throughout the hands and feet. The fingertip should be considered Topical diclofenac Oral celecoxib Limited duration of administration, with caution for adverse events Continuous patient education	Until development of HFS
Grade 1 treatment Minimal skin changes or dermatitis (e.g., erythema, edema, or hyperkeratosis) without pain	Observation until the symptoms worsen Same dose of chemotherapy should be considered Keep moisturizing and avoid stress 10% urea cream for exfoliation Continuous patient education	2 weeks If the symptom worsens, proceed to the next step
Grade 2 treatment Skin changes (e.g., peeling, blisters, bleeding, fissures, edema, or hyperkeratosis) with pain; limiting instrumental ADL	Very strong or strong class topical steroids. If symptom improves to grade 0 or 1, step down to medium class topical steroid Postponement until symptoms improve to grade 0 or 1 should be considered Dose reduction of chemotherapy should be considered Topical antibiotics if infection is suspected Continuous patient education Consultation with dermatologist	2 weeks If the symptom worsens or does not improve, proceed to the next step
Grade 3 treatment Severe skin changes (e.g., peeling, blisters, bleeding, fissures, edema, or hyperkeratosis) with pain; limiting self-care ADL	Topical strongest class steroids Treatment discontinuation or postponement until symptoms improve to grade 0 or 1 should be considered. If symptom improves to grade 0 or 1, step down to medium class topical steroid Dose reduction of chemotherapy should be considered Topical antibiotics if infection is suspected Continuous patient education Consultation with dermatologist	2 weeks

HFS, hand–foot syndrome; CTx, cytotoxic agent; CTCAE, Common Terminology Criteria for Adverse Events; ADL, activities of daily living

#### HFSR induced by MKIs

The only treatment with valid preventive effect evidence against HFSR is hydrocolloid dressings. Although a phase 3 randomized self-controlled study is ongoing [76], the evidence is currently insufficient. Therefore, future trials are warranted. Regarding HFSR treatment, topical steroids and antibiotics may be recommended, similar to CTx-related HFS. However, evidence-based recommendations cannot be made.

Table 6 summarizes possible management approaches. Management of HFSR induced by MKIs should be guided by CTCAE grading. Preventive measures (grade 0) require avoiding mechanical and chemical stress, limiting water exposure, use of appropriately fitted gloves, regular skin care, and continuous patient education. For grade 1 (mild) HFSR, same dose MKI treatment may be maintained. Supportive care includes moisturization, using 10% urea cream, and very strong or strong topical corticosteroids (to step-down to medium potency once improved). Grade 2 (moderate) HFSR, which restricts IADL, requires the strongest topical corticosteroids, potential treatment delay or dose reduction until improvement, and use of topical antibiotics if infection is suspected. Dermatology consultation is recommended, and step-down therapy should be applied once symptoms improve. For grade 3, (severe) HFSR, which interferes with self-care activities, necessitates the strongest class topical corticosteroids, chemotherapy interruption or discontinuation, and MKI dose reduction. Adjunctive measures include topical antibiotics for suspected infection, dermatology referral, and continuing patient education. Overall, patient education and proactive symptom management are emphasized across all grades to optimize adherence to MKI therapy.

**Table 6:** Management of HFSR induced by MKI

Grading of CTCAE	Management	Follow up
Grade 0 prevention	Avoid stress mechanical and chemical stress (excessive exercise and external damage) should be avoided Excessive exposure to water should be avoided and gloves are recommended Gloves that are tight in size are not recommended Moisturizing Skin care focusing on pressure sensitive areas Oral celecoxib Limited duration of administration, with caution for adverse events 10% urea cream Continuous patient education	Until development of HFS
Grade 1 treatment Minimal skin changes or dermatitis (e.g., erythema, edema, or hyperkeratosis) without pain	Same dose of chemotherapy should be considered Keep moisturizing and avoid stress 10% urea cream for exfoliation Topical very strong class or strong class steroids. If symptom is improved to grade 0, step down to medium class topical steroid Continuous patient education	2 weeks If the symptom worsens, proceed to the next step
Grade 2 treatment Skin changes (e.g., peeling, blisters, bleeding, fissures, edema, or hyperkeratosis) with pain; limiting instrumental ADL	Strongest class topical steroids If symptom improves to grade 0 or 1, step down to medium class topical steroid Treatment postponement until symptoms improve to grade 0 or 1 should be considered Dose reduction until symptoms improve to grade 0 or 1 may be considered Topical antibiotics in case infection is suspected Continuous patient education Consultation with dermatologist	2 weeks If the symptom worsens or does not improve, proceed to the next step
Grade 3 treatment Severe skin changes (e.g., peeling, blisters, bleeding, fissures, edema, or hyperkeratosis) with pain; limiting self-care ADL	Strongest class topical steroids If symptom improves to grade 0 or 1, step down to medium class topical steroid Treatment discontinuation or postponement until symptoms improve to grade 0 or 1 should be considered Dose reduction of MKI should be considered Topical antibiotics in case infection is suspected Continuous patient education Consultation with dermatologist	2 weeks

HFSR, hand–foot skin reaction; MKI, multi-kinase inhibitors; CTCAE, Common Terminology Criteria for Adverse Events; HFS, hand–foot syndrome; ADL, activities of daily living

#### Differences between Asia and other countries’ recommendations

No Asia-specific management guidelines for HFS/HFSR exist. High-potency steroid therapy is recommended for grades 1 and 2 HFS/HFSR in the United States and Europe [13]. However, topical steroids are not recommended for grade 1 HFS in Asian patients. While lidocaine patches and antiseptic solutions (e.g., 1% silver sulfadiazine) are recommended for grade ≥ 3 HFS in the West, evidence and safety data are lacking in Asia; thus, they are not being administered in clinics.

Differences between Asian and other race skin types should be considered. Asian skin has a thin stratum corneum (outermost layer) and dense cells, which are sensitive and may overreact when irritated [85, 86]. In contrast, Asian skin contains more melanin than Western skin, providing natural protection against ultraviolet radiation [87]. However, Westerners with lower melanin content are more prone to hyperpigmentation and may be more prone to 5-flurouracil-induced and steroid-induced hyperpigmentation. Management approaches should differ according to race and skin type.

#### Future research questions

In future, the prophylactic efficacy of diclofenac against capecitabine-induced HFS should be evaluated using a cream formulation as the base agent. Further evidence on the prophylactic efficacy of hydrocolloid dressing against MKI-induced HFSR is anticipated. A trial examining the efficacy of educational programs and multidisciplinary team approach should be conducted.

#### Limitations

This review has some limitations. This is not a systematic review, and quality assessment tools such as GRADE or the Cochrane risk-of-bias tool were not applied. In future, it is desirable to establish guidelines based on systematic review findings for patients in Asia, involving ore rigorous protocols. Despite its important region-specific recommendations, some of the suggested approaches, including the use of topical antibiotics, stress avoidance, and moisturizers, lack sufficient evidence to draw robust conclusions. Furthermore, the absence of ongoing trial results, including studies on diclofenac cream and topical steroids, limits our ability to make definitive recommendations. Additionally, there is a critical need for RCTs evaluating the effectiveness of educational programs and multidisciplinary team approaches in managing HFS and HFS/HFSR.

Further research based on differences in skin characteristics between Asian and Western populations is essential to develop treatment strategies that are both regionally appropriate and globally applicable. By addressing these gaps, future studies could contribute to improved patient outcomes and a more effective management of debilitating conditions.

## Conclusions

In this review, differences in skin biology between Asian and Western populations suggest the need for skin type-specific approaches to managing HFS/HFSR. No Asia-specific management guidelines for HFS/HFSR exist. High-potency steroid therapy is recommended for grade 1 and 2 HFS/HFSR in the United States and Europe. However, topical steroids are not recommended for HFS in Asian patients because of their different skin type. In addition, some medications, such as lidocaine patches or antiseptic solutions, used for grade ≥ 3, are not recommended for patients in Asia. In addition, diclofenac topical agents are expected to have a preventive effect on capecitabine-induced HFS. Recommendations considering the characteristics of Asian skin should be applied in clinical practice.

This review provides evidence-based recommendations from studies conducted on Asian patients, focusing on the prevention and treatment of HFS/HFSR. By addressing the unique skin characteristics of Asian populations, this review highlights the potential benefits of specific strategies such as the use of topical diclofenac and moisturizers. It also emphasizes the need for tailored approaches because differences between Asian and Western skin types necessitate Asian-specific management strategies that cannot be transferred directly from Western guidelines.

## Data Availability

The data supporting the findings of this study are available from the corresponding author, YI, upon reasonable request.
